# EXPLORING INTERNATIONAL CLASSIFICATION OF FUNCTIONING, DISABILITY AND HEALTH APPLICABILITY FOR CODING WORK-RELATED DISABILITY: A STUDY ON DEPRESSION AND FIBROMYALGIA IN SWEDISH SICK LEAVE CERTIFICATES

**DOI:** 10.2340/jrm.v56.36886

**Published:** 2024-11-21

**Authors:** Magdalena FRESK, Wilhelmus J. A. GROOTEN, Nina BRODIN, Lars G. BACKLUND, Britt ARRELÖV, Ylva SKÅNÉR, Anna KIESSLING

**Affiliations:** 1 Department of Neurobiology, Care Sciences and Society, Division of Family Medicine and Primary Care, Karolinska Institutet, Huddinge; 2 Department of Neurobiology, Care Sciences and Society, Division of Physiotherapy, Karolinska Institutet, Huddinge; 3 Women’s Health and Allied Health Professionals Theme, Medical Unit Occupational Therapy and Physiotherapy, Karolinska University Hospital, Stockholm; 4 Department of Orthopaedics, Division of Physiotherapy, Danderyd University Hospital, Stockholm; 5 Karolinska Institutet, Department of Clinical Sciences, Danderyd Hospital, Stockholm; 6 Department of Medical Specialities, Danderyd University Hospital, Stockholm, Sweden

**Keywords:** depression, disability evaluation, functional status, fibromyalgia, sick leave, social security, work capacity evaluation

## Abstract

**Objective:**

This study explores the effectiveness of using the International Classification of Functioning, Disability and Health (ICF) as a coding framework to document work-related disability information in sick leave certificates, focusing on depression and fibromyalgia in Sweden.

**Design:**

A qualitative ICF linking study was conducted, mapping information from 200 certificates per diagnosis to ICF.

**Methods:**

ICF linking rules were followed strictly. The coverage of ICF and ICF Core Sets was evaluated, proposing additional ICF categories when relevant categories were not included. Saturation of ICF categories was considered achieved if no new categories appeared in the last 5 certificates.

**Results:**

The study found high ICF coverage (85% for depression, 78% for fibromyalgia) in capturing work-related disability information. However, there was limited coverage in ICF Core Sets due to an excess of ICF categories in the Core Sets. Also, 2 additional relevant ICF categories for depression and 3 for fibromyalgia were identified.

**Conclusion:**

This study confirms that the International Classification of Functioning, Disability and Health is suitable for coding work-related disability in sick leave certificates. However, the identified limitations in ICF Core Sets highlights the need for context-specific subsets to enhance their relevance for depression and fibromyalgia in work-related disability.

## LAY ABSTRACT

The study investigates how Swedish physicians describe work-related disability in sick leave certificates, focusing on patients with depression and fibromyalgia. Currently, physicians use free text in sick leave certificates, making it challenging to analyse this information over time. This can affect fairness and equality in the social insurance system. The study examines whether the International Classification of Functioning, Disability and Health (ICF) can be a good way to code this information for depression and fibromyalgia. The results show that the ICF covers most of the needed codes for these conditions, but there are some gaps and also a surplus of non-relevant categories in the ICF Core Sets. Overall, the study concludes that the ICF is useful for information on work-related disability in sick leave, but the ICF Core Sets for depression and fibromyalgia need some context-specific adjustments to be useful.

When a patient has a sick leave period longer than a few weeks, which is often the case in depression and fibromyalgia, information regarding work-related disability is important for both sickness benefit and planning activities for rehabilitation and return to work (1). Yet, the data are difficult to capture for secondary use such as quality assurance purposes, which could hamper the possibility to analyse the data on work-related disabilities in social insurance over time and undermine legal certainty (2–4).

In Sweden, a sick leave certificate, containing information concerning the illness and issued by a physician, is typically required after 1 week of self-certification (5). The certificate is processed by the Swedish Social Insurance Agency (SSIA), and has a crucial role in determining the level of sickness benefits (6). The physician should not only describe the diagnosis in the sick leave certificate, but also outline the impairments of body functions and activity limitations caused by the disease (6). These descriptions should align with the aim and structure of the WHO International Classification of Functioning, Disability and Health (ICF) (6, 7). The patient’s diagnosis is presented in free text in the certificate and coded using ICD-10-SE. However, details regarding impairments of functioning related to work are currently conveyed in free text only.

The digitalization of healthcare has underscored the need for semantic interoperability to provide the best possible conditions for handling health data both in the clinical context and for secondary purposes. Health data standards are highlighted as important within the European Commission’s proposal for the regulation of a European Health Data Space (8) as well as in the WHO Global Strategy on Digital Health 2020–2025 (9). The WHO classification ICF was adopted in 2001 as a coding scheme for health information on functioning and disability, containing around 1,500 ICF categories (7). ICF is utilized worldwide but to varying extents in different countries (10). WHO has supported the process for developing ICF Core Sets –subsets of ICF categories relevant to specific health conditions or contexts – to enhance the implementation (11, 12). The last phase in the development of a new ICF Core Set includes validation, which should be conducted for different use-cases (13). According to a literature review in 2020, 35 ICF Core Sets for adults were developed following the suggested methodology between 2001 and 2019 (14). Twenty-three of them had been validated by 2019, with the majority using a qualitative Delphi method suggested by Grill et al. (13), although other methods such as ICF linking were also employed (14).

In a previous study we analysed the content validity of ICF and ICF Core Sets in the context of sick leave due to depression and long-term musculoskeletal pain (15). The ICF-linking method was applied on a relatively small sample of sick leave certificates from a medium-sized community in Sweden. The present study aims to expand the knowledge of the possible use of ICF and ICF Core Sets in sick leave, by using the same method on a large, national sample. Thus, the present study aims to validate to what extent the information in sick leave certificates regarding work-related disability due to depression and fibromyalgia can be linked to ICF and to relevant ICF Core Sets.

## METHODS

### Design and setting

A qualitative ICF linking study was conducted. Information on work-related disability in sick leave certificates issued for depression or fibromyalgia in Sweden was mapped to the ICF according to ICF linking rules (16–18). The comparative validity of ICF Core Sets was assessed by comparing the components of *body functions* and *activity and participation* in the ICF Core Sets with the information in the sick leave certificates. The ICF Core Set for depression (19) was compared with the ICF categories derived from the sick leave certificates issued for the diagnosis of depression, and the ICF Core Set for chronic widespread pain (20) was compared with the ICF categories derived from the sick leave certificates issued for the diagnosis of fibromyalgia.

### Data collection and study sample

In this study, we collected health data from sick leave certificates issued for depression (ICD-10 F32, F33) and fibromyalgia (ICD-10 M79.0 and M79.7) registered at the Swedish Social Insurance Agency (SSIA) for sickness cash benefit. If more than one sickness certificate was issued for the same patient during a cohesive sickness period, only the most recent one was included. The sample size of 200 certificates per diagnosis group was determined based on our previous study using the same method, but with a fourfold increase to ensure saturation (15).

The certificates were collected in 2 steps to secure that we reached the chosen sample size. Initially, 300 sickness certificates per diagnostic group, collected consecutively from 28 November 2017, based on dates of the first day of the sick leave period, were provided to the research group by SSIA. In the second step, these sick leave certificates were randomly ordered by the research group, using a random number generator (random.org). Each certificate was then checked manually by the research group to secure conformity of the ICD code and the diagnosis noted by the physician. However, this control did not lead to any exclusions. Thus, the first 200 certificates issued for depression and 200 certificates issued for fibromyalgia on the randomized list were included in the study.

### ICF linking

The ICF linking was performed by 1 physician and 2 physiotherapists in the research group, with expert knowledge of ICF and previous publications in the field. The first 5 certificates were linked by the group together and the remaining by at least 2 of the 3 group members. Each person performed their linking separately, filled in the linking protocol and presented the result at a decision meeting, and the final linking per item was agreed. If consensus was not reached at the decision meeting, the item was put on hold until additional information was gathered by each expert, and the item was then discussed again in the full group. If needed, a second opinion was provided by a fourth member of the research group, a physician and also psychologist, before the final decision was made. This procedure was repeated until consensus was reached for all items.

All information from the sections on functional impairments and activity limitations in each certificate was transferred verbatim to an ICF linking protocol, and the information was then separated into meaning units. In the next step, the concepts to be linked to ICF were identified by asking the question “What is this piece of information about’. The main and any additional concepts for each meaningful unit were identified and coded.

The ICF linking protocol contained the following information:

- serial and certificate number;
- meaningful unit, transferred verbatim from the certificate;
- aim of item (for clarification, when needed);
- main concept and additional concepts;
- preliminary linking of main and additional concepts;
- final linking of main and additional concepts.

### Codings

The coding followed the ICF linking rules, with a few remarks (16–18). If a concept could be linked to ICF, it was coded with the most precise ICF category. If a suitable ICF category could not be found, the concept was coded *not definable* with the additions general health; mental health, or physical health. If the concept was not included in the ICF, it was coded *not covered*, with the addition of a health condition when applicable. The term “codings” refers to all codes assigned in the ICF linking process, i.e., all endorsed ICF categories and the codes *not definable* and *not covered.* Also, if a meaning unit described the patient’s preserved functioning or any impairments or limitations (disability), it was not annotated. A case handled, but not specifically mentioned in the ICF linking rules, was if a meaning unit was not specific enough to choose an ICF category from only 1 of the components. In such cases we identified the main and additional concepts of the meaning unit and linked to both ICF components. An example was the meaning unit “focusing on”, which was linked to both the component of body functions “b140 Attention functions” (main concept) and “d160 Focusing attention” (additional concept). The different perspectives of health information (descriptive, appraisal, and needs or dependency) are mentioned in the refined ICF linking rules (18). The overall perspective of the health information in the sick leave certificates was descriptive, but the information was not detailed enough to specify the perspective any further.

### Saturation of data

Saturation of ICF categories was assumed to be reached if no new ICF category appeared in the last 5 sick leave certificates compared with those already linked.

### Comparison with ICF Core Sets for depression and chronic widespread pain

The ICF categories of the components of *body functions* and *activity and participation* derived from the ICF linking process were aggregated to the second level of the ICF classification hierarchy, for comparison with ICF Core Sets. The ICF categories were counted only once per certificate. An ICF category was defined as relevant for the context if it was identified in ≥ 10% of the certificates.

### Identified ICF categories not included in the ICF Core Sets

New ICF categories on the second level were presented as potential additional categories for this context if these were defined as relevant and were not included in the Comprehensive ICF Core Sets.

### Main outcome measures

The main outcome measures encompass the codings assigned during the ICF linking procedure. These codings encompass ICF categories or other health-related information that cannot be linked to the ICF.

### Data analyses

Data were analysed separately for the 2 diagnoses. The total number of meaning units and codings were described. Codings were categorized into 2 groups: (*i*) ICF categories and (*ii*) information not suitably codifiable with the ICF. The ratio of codings per group was computed. Furthermore, an analysis was conducted of the distribution of all endorsed ICF categories within the ICF components. The granularity of the ICF categories (second level or more detailed) was described. For a more detailed examination, ICF categories related to body functions and activity and participation were aggregated to the second level, with duplicates removed. The total sum of unique ICF categories across all certificates was calculated and presented per component. In preparation for the comparison and calculation of the overall coverage of the ICF Core Set, relevant ICF categories were selected. The overall coverage of the ICF Core Sets was defined as the number of relevant ICF categories derived from the sick leave certificates divided by all ICF categories in the Core Set. This method aligns with the approach employed in a preceding ICF validation study (21).

## RESULTS

### Sickness certificates issued for patients with a diagnosis of depression

*Overall codings*. The information was divided into 3,935 meaning units. A total of 3,935 main and 218 additional concepts were identified and these generated 4,153 codings (Table I). The main part of the codings (3,513/4,153, 85%) were within ICF, and 15% (640/4,153) were either not definable with or not covered by ICF (Table I). The codings within ICF (*n* = 3,513) were distributed mainly to *body functions* (2,263/3,513, 64%) and *activity and participation* (1,021/3,513, 29%).

**Table I:** Result of the ICF linking process of health data on work-related disability in sick leave certificates issued for depression and fibromyalgia

Factor	Depression	Fibromyalgia
Sickness certificates, *n*	200	200
Meaning units, *n*	3,935	4,922
Identified concepts		
Main concepts, *n*	3,935	4,922
Additional concepts, *n*	218	480
Total, *n*	4,153	5,402
*Codings assigned in the ICF linking process*		
*All codings of main and additional concepts, n (%)*		
ICF (all levels)	3,513 (85)	4,233 (78)
Not codable with ICF (not definable, not covered)	640 (15)	1169 (22)
Total	4,153 (100)	5,402 (100)
*Codings per ICF component, n (%)*		
Body functions	2,263 (64)	2,613 (62)
Body structures	2 (0)	137 (3)
Activity and participation	1,021 (29)	1,114 (26)
Environmental factors	227 (6)	369 (9)
Total	3,513 (100)	4,233 (100)
*Codings of “not codable with ICF’, n (%)*		
Not definable	152 (24)	498 (43)
Not covered	488 (76)	671 (57)
Total	640 (100)	1,169 (100)
*ICF categories derived from the sick leave certificates*		
*All ICF categories assigned, n (%)*		
Second level	1,725 (51)	1,998 (47)
Third level or more granular	1,785 (49)	2,232 (53)
Total	3,510 (100)	4,230 (100)
*Unique ICF categories on second level per ICF component, n (%)*		
Body functions	36 (38)	45 (42)
Body structure	2 (2)	10 (9)
Activity and participation	47 (49)	43 (40)
Environmental factors	11 (11)	9 (8)
Total	96 (100)	107 (100)

*Endorsed ICF categories.* A total of 1,725 ICF categories on the second level (51%) or a more granular level (49%) were assigned in the linking process (Table I). All 200 certificates contained information that was linked to at least 1 ICF category in the component of *body functions*, and 198 of the certificates contained information linked to at least 1 ICF category of activity and participation. The analyses of the ICF categories from the last 5 certificates showed that saturation was reached. When ICF categories were aggregated to the second level and duplicates were removed, 96 unique ICF categories remained. These categories were distributed to the components of *body functions* (*n* = 36), *activity and participation* (*n* = 47), *environmental factors* (*n* = 11), and *body structures* (*n* = 2). The vast majority (205/218) of the meaning units coded with 2 codes described aspects of “focusing on” and were coded with both “b140 Attention functions” (main concept) and “d160 Focusing attention” (additional concept).

*Confirmation of relevant ICF categories of body functions and activity and participation in the ICF Core Set for depression.* Thirteen of the unique second-level ICF categories for body functions and 11 for activity and participation were identified in ≥ 10% of the sick leave certificates and were mapped to the ICF Core Set for depression (Table II). Ten out of 13 (77%) ICF categories of body functions were identified in the Comprehensive ICF Core Set, and 5 out of 13 (38%) in the Brief ICF Core Set (Table II). For activity and participation, the corresponding figures were 9 out of 11 (82%) for the Comprehensive ICF Core Set and 3 out of 11 (27%) in the Brief ICF Core Set.

**Table II:** Unique ICF categories of the components of body functions and activity and participation derived from the sick leave certificates issued for depression ranked after relative frequency and compared with ICF Core Set for depression

Rank	Sickness certificates with the ICF category *n* (%)	ICF category		Included in the ICF Core Set		Not included in the ICF Core Set
Body functions				Comprehensive	Brief	
1	179 (89.5)	b152	Emotional functions	x	x	
2	158 (79.0)	b130	Energy and drive functions	x	x	
3	147 (73.5)	b140	Attention functions	x	x	
4	90 (45.0)	b134	Sleep functions	x		
5	71 (35.5)	b455	Exercise tolerance functions			x
6	69 (34.5)	b144	Memory functions	x		
7	68 (34.0)	b164	Higher-level cognitive functions	x		
8	39 (19.5)	b160	Thought functions	x		
9	37 (18.5)	b147	Psychomotor functions	x	x	
10	28 (14.0)	b280	Sensation of pain	x		
11	27 (13.5)	b126	Temperament and personality functions	x	x	
12	22 (11.0)	b114	Orientation functions			x
13	21 (10.5)	b230	Hearing functions			x
Activity and participation				Comprehensive	Brief	
1	149 (74.5)	d160	Focusing attention			x
2	105 (52.5)	d240	Handling stress and other psychological demands	x	x	
3	90 (45.0)	d850	Remunerative employment	x		
4	51 (25.5)	d710	Basic interpersonal interactions	x		
5	40 (20.0)	d740	Formal relationships			x
6	39 (19.5)	d210	Undertaking a single task	x		
7	30 (15.0)	d220	Undertaking multiple tasks	x		
8	27 (13.5)	d230	Carrying out daily routine	x	x	
9	26 (13.0)	d335	Producing nonverbal messages	x		
10	22 (11.0)	d640	Doing housework	x		
11	20 (10.0)	d177	Making decisions	x	x	

**Fig. 1:**
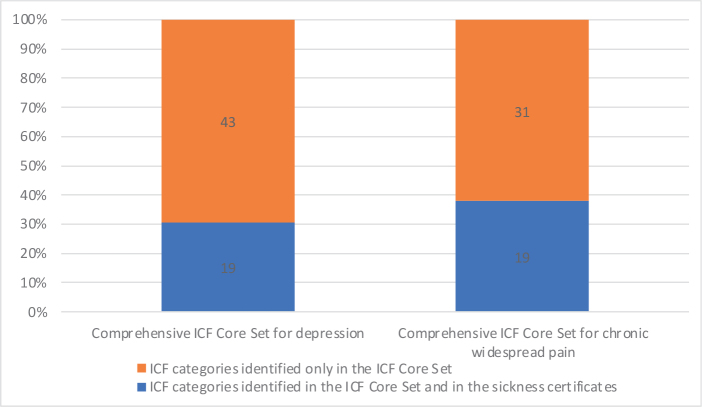
Illustration of the overall coverage of International Classification of Functioning Disability and Health (ICF) Core Sets for depression and chronic widespread pain: ICF categories of *body functions* and *activity and participation* derived from ≥ 10% of the sick leave certificates.

*Identified ICF categories not included in the ICF Core Set*. The overall coverage of the Comprehensive ICF Core Set was 30.1% (19/62), with 58.8% (10/17) in *body functions* and 20.0% (9/45) in *activities and participation* (Table III, Fig. 1). The overall coverage for the Brief ICF Core Set was 50% (8/16). From the concepts not covered in the Comprehensive ICF Core Set for depression, 5 ICF categories were identified as relevant but were missing in the Comprehensive ICF Core Set. These were: “b114 Orientation functions”, b230 “Hearing functions”, “b455 Exercise tolerance functions”, “d160 Focusing attention”, and “d740 Formal relationships” (Table IV).

**Table III:** Unique International Classification of Functioning, Disability and Health (ICF) categories in the components of *body functions* and *activity and participation*, identified in ≥ 10% of the sick leave certificates

Depression	Comprehensive ICF Core Set for depression			Brief ICF Core Set
	Body functions	Activity and participation	Total	Total
Number of ICF categories identified in the study	13	11	24	24
Number of ICF categories in the ICF Core Set	17	45	62	16
Number of ICF categories identified in the study and included in the ICF Core Set (% of all categories identified in the study)	10 (76.9)	9 (81.8)	19 (79.1)	8 (33.3)
Coverage of ICF Core Set from the study (%)	58.8	20.0	30.1	50.0
Fibromyalgia	Comprehensive ICF Core Set for chronic widespread pain			Brief ICF Core Set
	Body functions	Activity and participation	Total	Total
Number of ICF categories identified in the study	14	8	22	22
Number of ICF categories in the ICF Core Set	23	27	50	19
Number of ICF categories identified in the study and included in the ICF Core Set (% of all categories identified in the study)	11 (78.6)	8 (100)	19 (86.4)	12 (54.5)
Coverage of ICF Core Set from the study (%)	47.8	29.6	38.0	63.2

**Table IV:** International Classification of Functioning categories of the components of *body functions* and *activity and participation*, identified in more than 10% of the certificates and not included in Comprehensive ICF Core Sets

Identified ICF categories not included in Comprehensive ICF Core Sets		% of sick leave certificates
Depression		
d160	Focusing attention	74.5
b455	Exercise tolerance functions	35.5
d740	Formal relationships	20.0
b114	Orientation functions	11.0
b230	Hearing functions	10.5
Chronic widespread pain		
b144	Memory functions	28.5
b770	Gait pattern functions	16.5
b230	Hearing functions	14.0

### Sickness certificates issued for patients with a diagnosis of fibromyalgia

*Overall codings*. The information in the certificates was separated into 4,922 meaning units. A total of 4,922 main and 480 additional concepts were identified and generated 5,402 codings. The majority of the codings were within ICF (4,233/5,402, 78%) (Table I). Some 22% of the codings (1,169/5,402) were either not definable or not covered by ICF. The codings within ICF (*n* = 4,233) were distributed mainly to *body functions* (2,613/4,233, 62%) and to *activity and participation* (1,114/4,233, 26%).

*Endorsed ICF categories.* The information was linked to 1,998 ICF categories, almost equally distributed between the second level (47%) and the third level, or was more granular (53%) (Table I). All but 2 of the certificates contained information linkable to ICF categories of *body functions*, and 189 certificates contained information linkable to *activity and participation*. The analyses of the ICF categories from the last 5 certificates showed that saturation was reached. When the ICF categories were aggregated to the second level and duplicates were removed, 107 unique ICF categories remained. They were mainly distributed to the components of *body functions* (42%) and *activity and participation* (40%). The remaining ICF categories were distributed to *body structures* (9%) and *environmental factors* (8%). The certificates for fibromyalgia had 383 meaning units, which were coded with more than 1 code, and the majority (153/383, 40%) were descriptions of pain in a specific part of the body. This was coded with “b280 Sensation of pain” in combination with a code for the localization. The second largest group (141/383, 37%) were descriptions regarding aspects of “focusing on”, which was coded with both “b140 Attention functions” (main concept) and “d160 Focusing attention” (additional concept).

*Confirmation of relevant ICF categories of body functions and activity and participation in the ICF Core Set for chronic widespread pain.* Fourteen unique ICF categories of body functions, and 8 of activity and participation, were identified in ≥ 10% of the sick leave certificates and were mapped to the ICF Core Set for chronic widespread pain (Table V). Eleven out of 14 (78.6%) of the endorsed ICF categories of body functions were identified in the Comprehensive ICF Core Set and 6 out of 14 (42.9%) in the Brief ICF Core Set. For activity and participation, the corresponding figures were higher: 8 out of 8 for the Comprehensive ICF Core Set and 6 out of 8 (75%) for the Brief ICF Core Set.

**Table V:** Unique ICF categories of the components of *body functions* and *activity and participation* derived from sick leave certificates issued for fibromyalgia, ranked after relative frequency, and compared to ICF Core Set for chronic widespread pain

Rank	Sickness certificates with ICF category, *n* (%)	ICF category		Included in the ICF Core Set		Not included in the ICF Core Set
Body functions				Comprehensive	Brief	
1	193 (96.5)	b280	Sensation of pain	x	x	
2	153 (76.5)	b130	Energy and drive functions	x	x	
3	99 (49.5)	b152	Emotional functions	x	x	
4	98 (49.0)	b140	Attention functions	x		
5	93 (46.5)	b134	Sleep functions	x	x	
6	79 (39.5)	b710	Mobility of joint functions	x		
7	67 (33.5)	b455	Exercise tolerance functions	x	x	
8	57 (28.5)	b144	Memory functions			x
9	45 (22.5)	b730	Muscle power functions	x	x	
10	43 (21.5)	b164	Higher-level cognitive functions	x		
11	37 (18.5)	b265	Touch function	x		
12	33 (16.5)	b770	Gait pattern functions			x
13	28 (14.0)	b230	Hearing functions			x
14	26 (13.0)	b126	Temperament and personality functions	x		
Activity and participation				Comprehensive	Brief	
1	73 (37.0)	d850	Remunerative employment	x	x	
2	69 (35.0)	d240	Handling stress and other psychological demands	x	x	
3	64 (32.0)	d410	Changing basic body position	x		
4	55 (28.0)	d415	Maintaining a body position	x		
5	49 (25.0)	d640	Doing housework	x	x	
6	40 (20.0)	d430	Lifting and carrying objects	x	x	
7	31 (16.0)	d450	Walking	x	x	
8	27 (14.0)	d230	Carrying out daily routine	x	x	

*Identified ICF categories not included in the ICF Core Sets.* The overall coverage of the Comprehensive ICF Core Set was 38.0%, with 47.8% in body functions and 29.6% in activities and participation (Table III, Fig. 1). Three ICF categories were identified as relevant for the diagnosis of fibromyalgia in this context but were not included in the Comprehensive ICF Core Set for chronic widespread pain (Table IV). These were: “b144 Memory functions”, “b770 Gait pattern functions”, and “b230 Hearing functions”.

## DISCUSSION

The results of the study show that ICF covers the majority of the information on work-related disability in sick leave certificates, with a level of ICF-linked meaning units of 85% for depression and 78% for fibromyalgia. Also, the comparison between the ICF categories identified in the sick leave certificates and the ICF Core Sets revealed that the coverage was low, indicating that the ICF Core Sets for both diagnoses contain a surplus of ICF categories that are not necessarily relevant for this context. Finally, some additional ICF categories for depression (exercise tolerance functions and hearing functions) and for fibromyalgia (memory functions, gait pattern functions, and hearing functions) were identified as important for the context of sick leave, although not included in the ICF Core Sets.

### Findings in relation to other studies

*ICF-coded health data on work-related disability in sick leave certificates in relation to the content of ICF.* All certificates had information linkable to ICF categories of body functions and activity and participation, confirming an overall improvement in the documentation of information on functional impairments and activity limitations in Swedish sick leave certificates, as suggested in previous studies in the field (4, 15). The rate of meaningful units linkable to ICF categories was nearly identical to our previous ICF-linking study that employed the same methodology on a smaller sample of sickness certificates issued for depression and long-term musculoskeletal pain (15). However, the number of uniquely identified ICF categories almost doubled in this study compared with the previous one, likely attributable to the larger national sample size in this study.

### ICF coded health data on work-related disability in the sick leave certificates in relation to ICF Core Sets

*Comprehensive ICF Core Set for depression.* The results confirm that the ICF categories derived from certificates issued for depression were well covered by the Comprehensive ICF Core Set for depression, consistent with our previous study (15). Additionally, 5 ICF categories were initially identified as potential additions for work-related disability information based on their relevance ranking, but after a more thorough analysis, only 2 of them (exercise tolerance functions and hearing functions) were deemed relevant (Table V). The most frequently identified potential additional ICF category was “b455 Exercise tolerance functions”, present in 35.5% of the certificates. The concepts mapped to the code were fatiguability and reduced physical endurance. Fatigue is one of the DSM5 criteria for depression (22). Also, “b455 Exercise tolerance functions” is included in the ICF Core Set for Disability Evaluation in Social Security, supporting the relevance of adding this ICF category when describing work-related disability (23). The ICF category “b230 Hearing functions” was also identified as a potential additional ICF category for this context, but just above the arbitrary threshold of 10% relative frequency (10.5%). The prevalent concept associated with “b230 Hearing functions” in the majority of certificates was “sound sensitivity”, a symptom well described in association with depression. As “sound sensitivity”“ lacks a specific ICF category, discussions arose during the linking process. However, we agreed to link the concept to “b230 Hearing functions” to maintain consistency with the logic of the classification hierarchy, given that “light sensitivity” is placed under “b210 Seeing functions”. It could be argued that the concept should be linked to “b240 Sensations associated with hearing and vestibular functions”, and future updates of the classification may benefit from a specific ICF category or inclusion for “sound sensitivity”. According to the relevance ranking of the ICF categories, “b114 Orientation functions” (11%) and “d740 Formal relationships” (20%) were also considered as potential additions. However, as the concepts behind them described normal functioning in all cases, they were deemed irrelevant for this context. The ICF category “d160 Focusing attention” was also identified as a potential additional category but is discussed separately below.

*Brief ICF Core Set for depression.* The Brief ICF Core Set is anticipated to encompass the most central ICF categories for the given diagnosis, demonstrating high conformity with the identified relevant ICF categories. However, a few noteworthy observations should be considered. We noted that sleep and memory functions were frequently described in a significant number of certificates (45% and 34.5% respectively), yet they are not included in the Brief ICF Core Set. Additionally, “d710 Basic interpersonal interactions” was present in a quarter of all certificates. Interpersonal interactions have been recognized for their importance in workability (23) and this category was identified as relevant in our previous study (15). Thus, these categories should be regarded as relevant additions to the Brief ICF Core Set, if utilized as a subset in this context.

*Comprehensive ICF Core Set for chronic widespread pain.* The ICF categories derived from the certificates issued for fibromyalgia were well captured by the Comprehensive ICF Core Set for chronic widespread pain. Nevertheless, 3 ICF categories deemed relevant for work-related disability in sick leave due to fibromyalgia were identified but are not included in the Comprehensive ICF Core Set: memory functions, gait pattern functions, and hearing functions. The most frequently occurring potential additional ICF category was “b144 Memory functions”, present in 28.5% of the certificates. Memory function was also identified as a potential additional ICF category in the validation study of the ICF core set for patients with fibromyalgia by Hieblinger et al. (24). Cognitive problems such as memory loss in patients with fibromyalgia is well described in the literature and the ICF category should be considered as a relevant addition to the ICF Core Set for use in this context (25). Additionally, information on limping gait, and stiff or slow movement patterns, was present in 16.5% of the certificates and was linked to “b770 Gait pattern functions”. This ICF category was also identified as relevant in the ICF Core Set validation study by Hieblinger et al. (24). Finally, similar to sickness certificates issued for depression, information regarding vertigo, tinnitus, and sound sensitivity was present in 14% of the sickness certificates and linked to “b230 Hearing functions”. Depending on work duties the ICF category also seems to be a relevant addition for fibromyalgia, and adding an inclusion to the ICF category in the classification would help the coding to conform.

Several categories of *body functions* and *activity and participation* within the Comprehensive ICF Core Set for chronic widespread pain were not identified as relevant in this study. Although these ICF categories have been confirmed from the patient’s perspective in fibromyalgia in the validation study by Hieblinger et al. (24), they seem not be central for describing impairments of functional impairments and activity limitations related to work.

*Brief ICF Core Set for chronic widespread pain.* he most common ICF category not included in the Brief ICF Core Set was "b140 Attention functions", which is due to the methodology and discussed under a separate heading. The second most common ICF category (39.5% relevance ranking) not included in Brief ICF Core Set was "b710 Mobility of joint functions". The concept behind it was, in most cases, joint stiffness. According to the high relevance ranking, it might be a relevant addition also to the Brief ICF Core Set when used in this context.

*Focusing attention and attention functions and remunerative employment.* A special case was the most common ICF category for both diagnoses not included in either of the ICF Core Sets, “d160 Focusing attention”, as the high frequency could be explained by our methodology. We used 2 codes for meaning units regarding “focusing on” when the background information did not support only 1 of the components. The result was a high frequency for both “b140 Attention functions” (included in ICF Core Set) and “d160 Focusing attention” (not included in ICF Core Set). The overlap between “b140 Attention functions” and “d160 Focusing attention” and the difficulties in distinguishing between the categories is well described by other researchers (26–28). Thus, we do not find it necessary to add “d160 Focusing attention” for this context, and in future ICF linking it would be more practical to choose only “b140 Focusing attention” for the concept “focusing on” in case information is not available to support the component of *activity and participation.*

Also worth mentioning is the high relative frequency of “d850 Remunerative employment” for both diagnoses. The information linked to this ICF category described the person’s situation in relation to work, for example work hours, and work-related duties. This information is expected in a sick leave certificate addressing both SSIA and the employer but, as in our previous study (15), we do not find this information well captured by only 1 ICF category.

### Methodological considerations

We consider the sample to be representative of information on work-related disability for depression and fibromyalgia in Sweden. Utilizing a consecutive national sample of 200 sickness certificates per diagnosis, our study features a substantial sample size compared with other similar studies in the field. Additionally, saturation of ICF categories was achieved for both diagnoses. Our study specifically focused on the ICF components of *body functions* and *activity and participation*. While contributing valuable insights into work-related disability during sick leave, it is important to note that our study does not constitute a full validation of the ICF Core Sets. However, the researchers conducting the linking procedure in this study are ICF experts with physician or physiotherapist professional skills and had previously conducted several ICF linking studies (15, 29). Strict adherence to ICF linking rules guaranteed the quality of the linking process. Triangulation was applied through the formation of a multidisciplinary linking team, the examination of 2 diagnoses, and the confirmation of results in relation to both the Comprehensive and the Brief Core Sets. Taking these factors into account, there remains a potential for variations in the selection of endorsed ICF categories by other coders, and there remains a possibility that relevant ICF categories were not included. Finding and comparing previous linking in the linking process was also challenging due to the various methods of presenting findings in peer-reviewed journals.

### Implications

Given that the overall coverage for both diagnoses did not exceed 50%, a smaller subset of ICF categories may be adequate for this context, at least as an initial selection. This subset could be derived from the ICF categories identified as relevant in our study, incorporating the proposed additional categories. The suggested additions to the ICF Core Sets, both in our study and in previous research by others, also prompt questions about how to ensure the ongoing accuracy of the ICF Core Sets. Addressing this issue constitutes a substantial and crucial task that necessitates attention in the near future.

### Conclusions

In conclusion, our findings support the feasibility of the International Classification of Functioning, Disability and Health (ICF) as a coding scheme for information on work-related disability in sick leave certificates. This system effectively covers a major proportion of the information provided by the physician. However, the analysis comparing the Comprehensive ICF Core Sets with the ICF categories derived from the certificates revealed limited coverage. This indicates an excess of ICF categories within the ICF Core Sets for this context. Additionally, our study identifies 2 ICF categories associated with depression that are not included in the existing ICF Core Sets, and proposes their incorporation for the use-case of work-related disability. Furthermore, we suggest the inclusion of 3 additional categories for fibromyalgia as meaningful enhancements, also for this context.
